# Role of Xingnaojing Injection in treating acute cerebral hemorrhage: A systematic review and meta-analysis

**DOI:** 10.1097/MD.0000000000019648

**Published:** 2020-04-10

**Authors:** Xiao Ma, Tao Wang, Jianxia Wen, Jian Wang, Nan Zeng, Wenjun Zou, Yuxue Yang

**Affiliations:** School of Pharmacy, Chengdu University of Traditional Chinese Medicine, Chengdu, China.

**Keywords:** acute cerebral hemorrhage, meta-analysis, randomized controlled trials, systematic review, xingnaojing injection

## Abstract

**Background:**

Xingnaojing injection (XNJi) is widely used for acute cerebral hemorrhage. However, the efficacy of XNJi for acute cerebral hemorrhage has not been comprehensively proved by systematic analysis yet. Therefore, it is essential to evaluate the efficacy and safety of XNJi in an evidence-based method.

**Methods:**

Six databases were searched with XNJi used for acute cerebral hemorrhage in randomized controlled trials (RCTs). Meta-analysis was performed by Review Manager 5.3. The efficacy rate, brain edema, cerebral hematoma, neurological deficit score, hs-crp, Glasgow Coma Scale (GCS), and activities of daily living (ADL) were systematically evaluated. The Cochrane risk of bias was used to evaluate the methodological quality of eligible studies.

**Results:**

This study is registered with PROSPERO (CRD42018098737). Twenty-nine studies with a total of 2638 patients were included in this meta-analysis. Compared with conventional treatment, XNJi got higher efficacy rate (OR = 3.37, 95% CI [2.65, 4.28], *P* < .00001). Moreover, XNJi showed significant enhancement of efficacy rate via subgroup analysis in course and dosage. In addition, XNJi demonstrated significant improvement in Chinese stroke scale (CSS) and National Institutes of Health Stroke Scale (NHISS) (mean difference [MD] = −4.74, 95% CI [−5.89, −3.60], *P* < .00001; MD = −4.45, 95% CI [−5.49, −3.41], *P* < .00001), GCS (MD = 2.72, 95% CI [2.09, 3.35], *P* < .00001). It also remarkably decreased the level of hs-crp (MD = −6.50, 95% CI [−7.79, −5.21], *P* < .00001), enhanced ADL (MD = 20.38, 95% CI [17.98, 22.79], *P* < .00001), and alleviated hematoma and edema (MD = −2.53, 95% CI [−4.75, −0.31] *P* < .05; MD = −1.74 95% CI [−2.42, −1.07] *P* < .00001) compared with conventional treatment.

**Conclusion:**

XNJi is effective in treating acute cerebral hemorrhage with significant improvement of CSS, NHISS and impairment of hs-crp, hematoma, and edema compared with conventional treatment. Moreover, XNJi got remarkable efficacy at the dose of 20, 30, 60 mL and from 7 to 28 days. No serious adverse reactions occurred. These results were mainly based on small-sample and low-quality studies. Therefore, more rigorous, large-scale RCTs were further needed to confirm its efficacy, safety, and detailed characteristic of application.

## 1 Introduction

Stroke is the second most common cause of death and the leading cause of disability all over the world. There is an especially tremendous impact on middle-income countries in a few decades. As in China, stroke is already the leading cause of adult disability and death.^1^,^2^ Acute cerebral hemorrhage is one of the important causes of stroke.^[3]^ The number of patients with acute cerebral hemorrhage is also accordingly increasing with the risk factors such as hypertension and diabetes. At present, acute cerebral hemorrhage is believed to be the intractable problem in clinic. However, there is still no ideal therapy available.^[4]^ Nowadays, several conventional therapies were commonly used in acute cerebral hemorrhage treatment. In that, neuroprotective agents such as edaravone were the main kind of medicine for acute cerebral hemorrhage.^[5]^ However, several researches reported that edaravone treatment might got controversial result. Moreover, it might also cause renal dysfunction, disseminated intravascular coagulation (DIC), and even irreversible multiple organ failure (MOF).^[6]^ Therefore, finding new agents for acute cerebral hemorrhage is urgently needed.

Traditional Chinese medicine (TCM) has been used as complementary therapy for acute cerebral hemorrhage for decades. Among these, Xingnaojing injection (XNJi) is one of the most common used traditional Chinese patent medicines for acute cerebral hemorrhage treatment. In recent years, XNJi accompany with decreasing blood pressure, maintaining water and electrolyte balance, and neuroprotective agent therapy is thought as effective at acute stage of acute cerebral hemorrhage in China. It can significantly enhance the efficacy and decrease the complications according to the majority reports of literatures. XNJi is comprised of multiple Chinese materia medica such as Musk, Synthetic Borneol, *Curcuma aromatica* Salisb, and *Gardenia jasminoides* Ellis. It got various effects such as resuscitation, antipyretic action, activating blood circulation, cooling blood, and eliminating toxins.^[7]^ Recent research reported that XNJi could penetrate blood brain barrier (BBB) and directly act on the central nervous system.^[8]^ In addition, the effect of XNJi on alleviating hydrocephalus, scavenging free radicals, promoting patient recovery, shortening coma time, and reducing complications were believed to improve the function of BBB permeability and benefit in acute cerebral hemorrhage.^9^,^10^

There were abundant reports regarding XNJi as an available treatment measure for acute cerebral hemorrhage. However, systemic evaluation on its therapeutic effects is lacking. Nowadays, more and more TCM is gradually re-confirmed via systemic review method as trends in China. Thus, in order to assess the application value of XNJi on acute cerebral hemorrhage, a systemic analysis was carried to concern its efficacy and safety.

## 2 Materials and methods

### 2.1 Search strategy

This systematic review had been registered in PROSPERO and the registration number is CRD42018098737. The databases included China National Knowledge Infrastructure (CNKI), VIP medicine information system (VMIS), Wanfang, Embase, PubMed, and Cochrane Library. The dates ranged from the establishment to August 2017. In our study, “P” should be “acute cerebral hemorrhage.” “I” should be “Conventional treatment (including lowing blood pressure, maintaining water and electrolyte balance, and neuroprotective agent).” “C” should be “Xingnaojing injection with or without conventional treatment.” “O” should be “efficacy rate.” However, the range of conventional treatment is so wide. In addition, the name of Xingnaojing injection is the specific name. Therefore, the following initial search items were used: “Xingnaojing injection” [Title/Abstract] and “acute cerebral hemorrhage” [Title/Abstract] or “hemorrhagic stroke” [Title/Abstract] in both Chinese and English. The searched results were downloaded for the further screening.

### 2.2 Inclusion criteria

The inclusion criteria were as follows: all randomized controlled trials (RCTs) of XNJi were included. Treatment group was the conventional treatment combined with XNJi, whereas control group was conventional treatment alone. Acute cerebral hemorrhage was diagnosed according to definite diagnostic criteria and CT/MRI. The age and sex of patients were not restrictive.

### 2.3 Exclusion criteria

The exclusion criteria were as follows: repeated published literature. Studies with incomplete or incorrect data. Patients with cerebral infarction and severe organ dysfunction. Treatment group or control group combined with other TCM during treatment. Animal experiments and review literatures.

### 2.4 Data extraction

The general information, including diagnostic criteria, interventions, outcome measures, and adverse reaction were extracted by 2 researchers (TW and YXY) independently. The extracted data were showed as following: general information, including first author, published year, the number of participants in treatment and control group respectively. Intervention, including the dosage, treatment course, and the combining drugs of XNJi were also extracted. Outcome measures, including efficacy rate, brain edema, cerebral hematoma, neurological deficit score, hs-crp, Glasgow coma scale (GCS), and activities of daily living (ADL) were recorded for further analysis. This research was based on synthesizing clinical trials’ data and it would not leak out patients’ information. Therefore, ethical approval for this research is unnecessary to be conducted.

### 2.5 Quality assessment

The quality of literature was evaluated according to the following items: random sequence generation, allocation concealment, blinding of participants and personnel, blinding of outcome assessment, incomplete outcome date, selective reporting, and other bias. Each item was assessed using the 3 levels of “low,” “high,” and “unclear.” The retrieval process and quality evaluation in accordance with the above items were carried out by 2 reviewers independently, and cross checked (TW and JXW). Discussion would be carried out if any differences generated.

### 2.6 Statistical analysis

RevMan5.3 software (Cochrane Collaboration, Oxford, UK).provided by Cochrane Collaboration was utilized for meta-analysis. Odds ratio (OR) was adopted in dichotomous variable, such as efficacy rate. Meanwhile, mean difference (MD) was applied in continuous variables, such as neurological deficit score, coma index score, hematoma volume, edema volume, and hs-crp. Both OR and MD were expressed with 95% CI. *I*-square (*I* ^2^) and *P*-value were used to evaluate heterogeneity. Fixed effect model was adopted for meta-analysis in the case of no significant heterogeneity (*P* ≥ .1, *I* ^2^ ≤ 50%) and the total OR value or MD value and 95% CI were calculated. Random effect model was adopted for meta-analysis in the case of substantial heterogeneity among studies (*P* < .1, *I* ^2^ > 50%). Subgroup analysis investigated the effect of various administration doses as well as administration courses of treatment on efficacy rate. The funnel plot was adopted to analyze the publication bias of enrolled researches.

## 3 Results

### 3.1 Inclusion of studies

**Figure 1:**
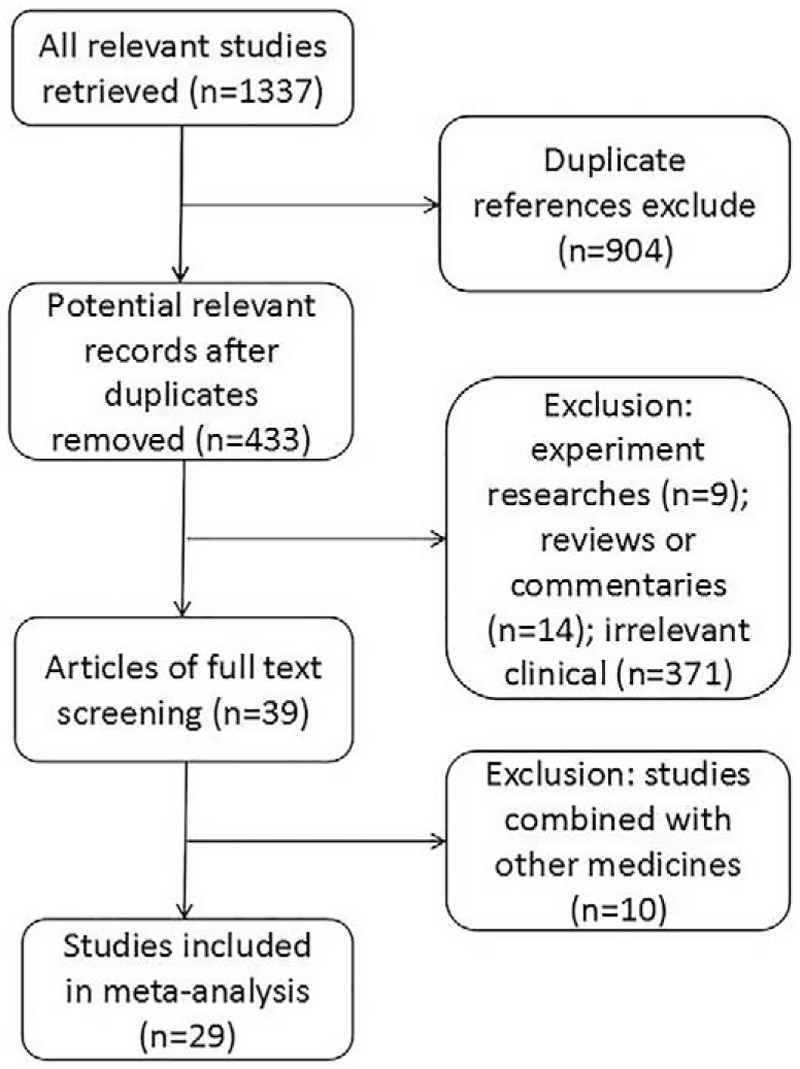
Flowchart of studies selection.

A total of 1337 articles were retrieved according to the search strategy. After the title and abstracts screening, the studies including the duplicate reviews, animal experiments, reviews were excluded. After further reading, 10 studies combined with other medicines were excluded. Twenty-nine studies were eventually included in this meta-analysis (Fig. 1).^11^,^12^,^13^,^14^,^15^,^16^,^17^,^18^,^19^,^20^,^21^,^22^,^23^,^24^,^25^,^26^,^27^,^28^,^29^,^30^,^31^,^32^,^33^,^34^,^35^,^36^,^37^,^38^,^39^

### 3.2 Characteristics of the included studies

All the 29 studies were designed as XNJi combined with conventional treatment versus conventional treatment (Table 1). A total of 2638 patients were included in this meta-analysis. Patients with acute cerebral hemorrhage diagnosed according to definite criteria and CT/MRI were included. The dose of XNJi varied from 20 to 60 mL and the course of XNJi ranged from 7 to 28 days. Conventional treatment included application of mannitol to reduce intracranial pressure, neurotrophic drugs, and antihypertensive drugs as well as prevention of infection and other symptomatic treatment.

**Table 1:**
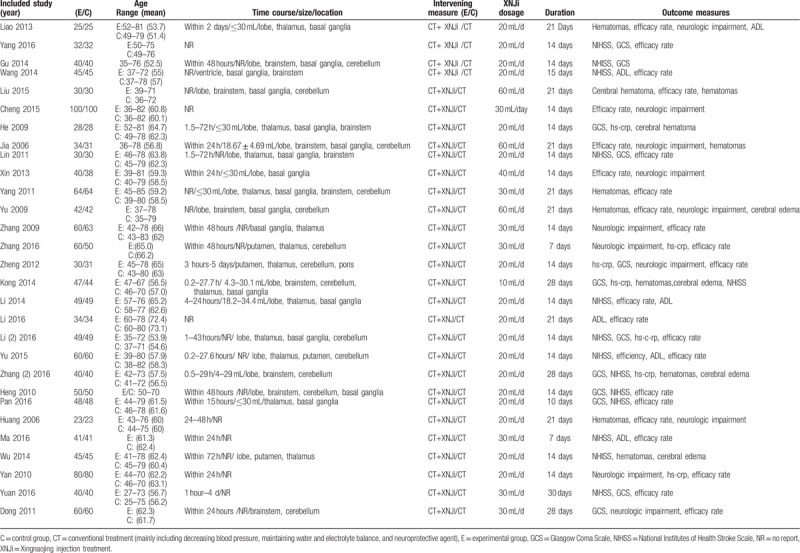
Characteristics of included studies.

### 3.3 Quality of study

None of the studies indicated whether the blind method and randomized hiding were used. Six studies carried out the research by a random number table allocation method.^22^,^23^,^24^,^26^,^27^,^28^ However, the remaining studies mentioned randomized method but did not explain the specific random grouping. All studies did not mention whether a hidden allocation was performed. None of the studies reported blinding of participants and personnel or blinding of outcome assessment. Moreover, the incomplete outcome data were low in all the studies. In addition, 2 studies^11^,^36^ got high risk of selective reporting and other studies were relatively low in this bias. There were unclear risks of bias in all the studies (Table 2).

**Table 2:**
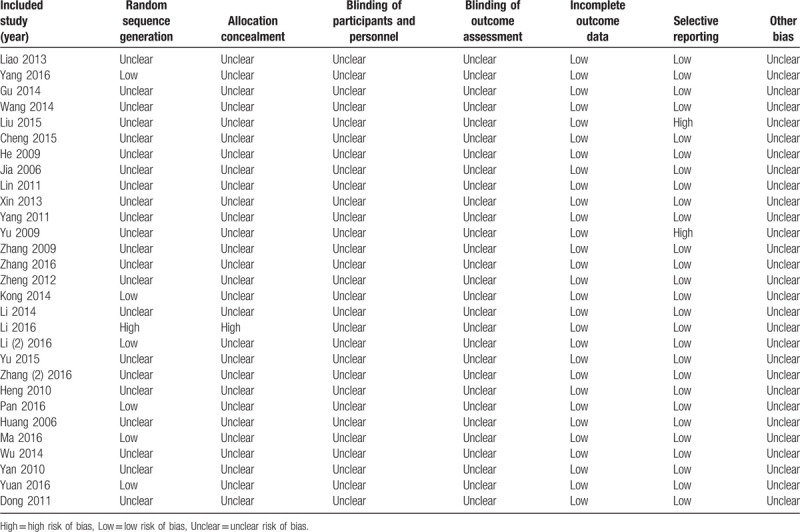
Risk of bias summary.

### 3.4 Results of efficacy and safety analysis

#### 3.4.1 Efficacy rate

**Figure 2:**
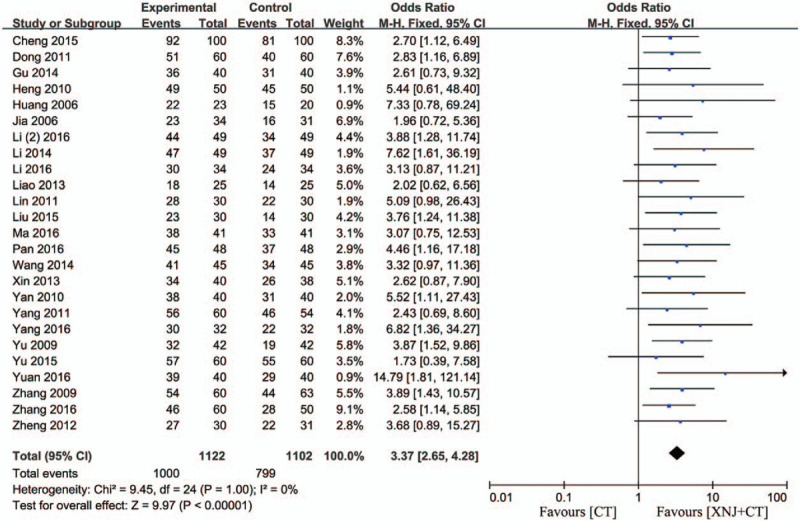
Efficacy rate of XNJi combined with conventional treatment versus conventional treatment. *I* ^*2*^ and *P* are the criterion for the heterogeneity test, ♦ pooled odds ratio, —▪— odds ratio and 95% CI.

Twenty-five studies reported the efficacy rate according to the “stroke in the 4th National Cerebrovascular Disease Conference”.^11^,^12^,^13^,^15^,^16^,^17^,^18^,^19^,^20^,^21^,^22^,^24^,^26^,^27^,^28^,^29^,^30^,^31^,^32^,^33^,^34^,^35^,^36^,^37^,^38^ There was no heterogeneity (*P* = 1.00, *I* ^*2*^ = 0%), and the fixed-effect model was used to carry out the meta-analysis. The result demonstrated that compared with the conventional treatment, XNJi could significantly increase the efficacy rate of patients with acute cerebral hemorrhage (OR = 3.37, 95% CI [2.65, 4.28] *P* < .00001) (Fig. 2).

#### 3.4.2 Efficacy rate of XNJi in different courses and dosage

**Figure 3:**
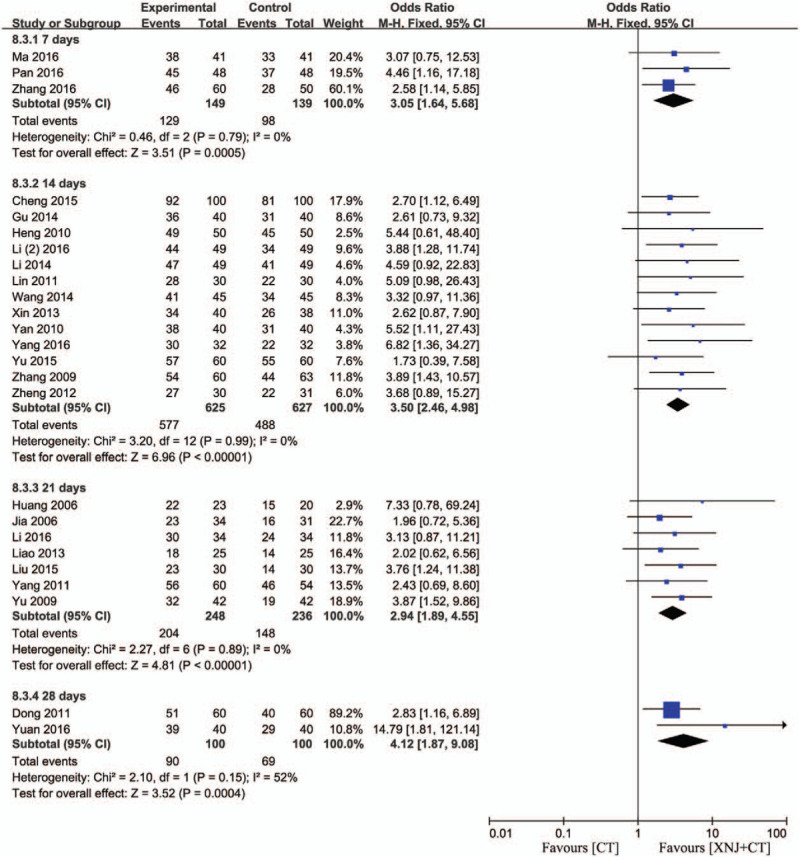
Efficacy rate of XNJi in different courses. *I* ^*2*^ and *P* are the criterion for the heterogeneity test, ♦ pooled odds ratio, —▪— odds ratio and 95% CI.

Three studies included the treatment for 7 days,^24^,^28^,^37^ 13 studies included the course for 14 days^13^,^15^,^16^,^18^,^19^,^22^,^26^,^27^,^30^,^33^,^34^,^36^,^38^ and 7 studies included the course for 21 days.^11^,^12^,^20^,^21^,^31^,^32^,^35^ Moreover, 2 studies recorded the course for 28 days.^17^,^29^ There was no substantial heterogeneity in these 4 subgroups (*P* = .79, *I* ^*2*^ = 0%; *P* = .99, *I* ^*2*^ = 0%; *P* = .89, *I* ^*2*^ = 0%; *P* = .15, *I* ^*2*^ = 52%), and the fixed-effect model was carried out. These results demonstrated that the efficacy rate of XNJi during 4 courses was significantly higher than that of conventional treatment respectively (OR = 3.05, 95% CI [1.64, 5.68] *P* = .0005; OR = 3.50, 95% CI [2.46, 4.98] *P* < .00001; OR = 2.94, 95% CI [1.89, 4.55] *P* < .00001; OR = 4.12, 95% CI [1.87, 9.08] *P* = .0004) (Fig. 3).

**Figure 4:**
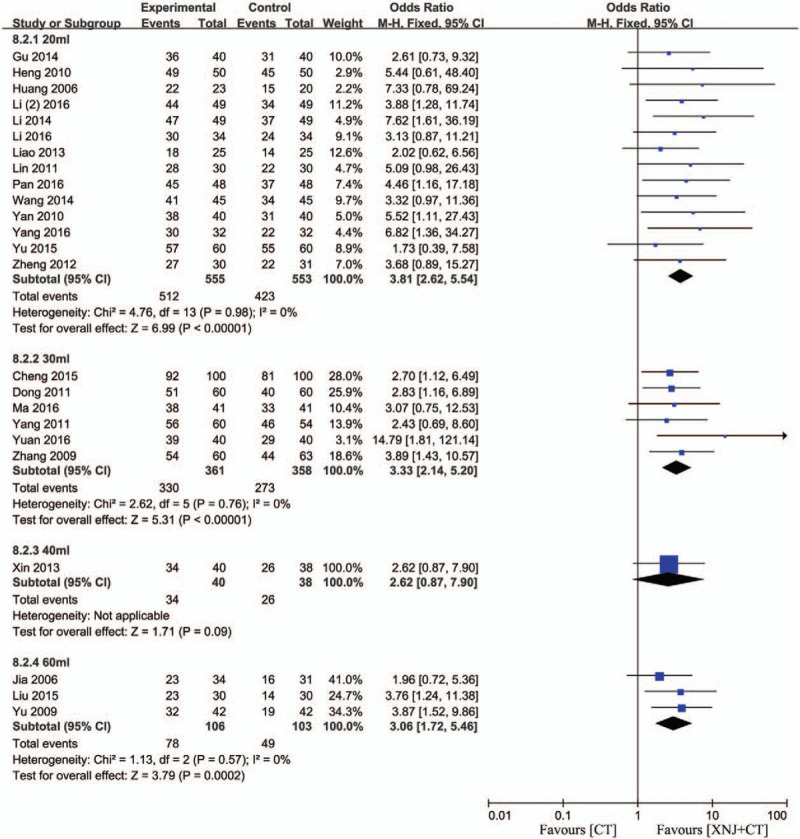
Efficacy rate of XNJi in different dosage. *I* ^*2*^ and *P* are the criterion for the heterogeneity test, ♦ pooled odds ratio, —▪— odds ratio and 95% CI.

Additionally, subgroup analysis was carried out to investigate the influence of dosage. According to the dosage, there primarily existed 20 mL,^12^,^13^,^18^,^19^,^21^,^22^,^26^,^27^,^28^,^31^,^33^,^36^,^38^ 30 mL,^15^,^16^,^17^,^24^,^29^,^32^,^34^,^37^ 40 mL,^[30]^ and 60 mL^11^,^20^,^35^ groups. There was no substantial heterogeneity in 20, 30, and 40 mL subgroups (*P* = .98, *I* ^2^ = 0%; *P* = .76, *I* ^2^ = 0%; *P* = .57, *I* ^2^ = 0%). The above data and analysis showed that the efficacy rate of XNJi with 20, 30, and 60 mL were significantly higher than conventional treatment (OR = 3.81, 95% CI [2.62, 5.54] *P* < .00001; OR = 3.33, 95% CI [2.14, 5.20] *P* < .00001; OR = 3.06, 95% CI [1.72, 5.46] *P* = .0002). However, there was no difference of efficacy rate between XNJi in 40 mL and conventional treatment (OR = 2.62, 95% CI [0.87, 7.90] *P* = .09) (Fig. 4).

#### 3.4.3 Adverse reactions

A total of 6 studies reported adverse events.^16^,^23^,^26^,^28^,^35^,^36^ Among them, there were 17 cases of adverse reaction in the treatment group, including emesis, skin rash, diarrhea, and chest tightness. In the control group, there were 22 cases of adverse reaction events including emesis, diarrhea, nausea, somnolence, and tachycardia (Table 3). All these adverse reactions disappeared after withdrawal of intervention.

**Table 3:**
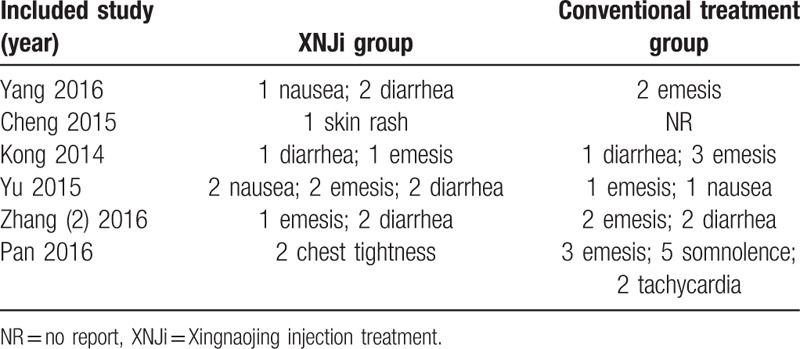
The adverse events in XNJi and conventional treatment.

### 3.5 Neural functional deficit score

**Figure 5:**
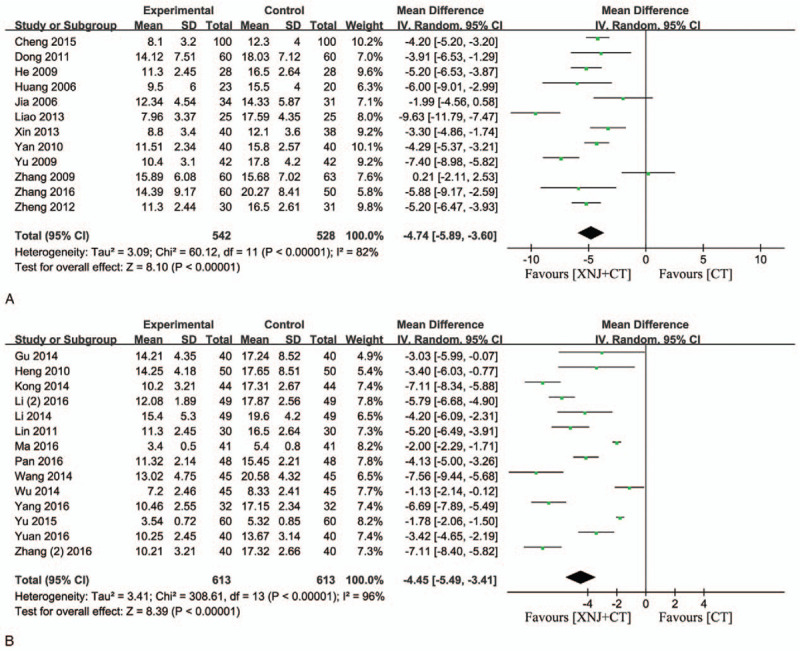
Improvement of neurological deficit score. (A) The forest plot of score of CSS. (B) The forest plot of evaluation of nerve function defect in accordance with NIHSS. *I* ^*2*^ and *P* are the criterion for the heterogeneity test, ♦ pooled mean difference, —▪— mean difference and 95% CI. CSS = Chinese stroke scale, NIHSS = National Institutes of Health Stroke Scale.

Twenty-six studies reported the neural functional deficit score. The *I*-square (*I* ^*2*^) statistic indicated that there was significant heterogeneity among 26 trials (National Institutes of Health Stroke Scale [NIHSS], *I* ^2^ = 96%, *P* < .00001; GCS, *I* ^2^ = 82%, *P* < .00001), and random-effect model was used to pool the results of these trials.^13^,^14^,^15^,^16^,^17^,^18^,^19^,^20^,^21^,^22^,^23^,^24^,^25^,^26^,^27^,^28^,^29^,^30^,^31^,^33^,^34^,^35^,^36^,^37^,^38^,^39^ Twelve studies were based on the score of Chinese stroke scale (CSS, the fourth national cerebrovascular disease meeting about the degree of neurological impairment standard neurological deficit scores in 1995).^15^,^16^,^17^,^20^,^21^,^30^,^31^,^34^,^35^,^37^,^38^,^39^ The result showed that compared with conventional treatment, XNJi significantly improved the neurological function in patients with acute cerebral hemorrhage (MD = −4.74, 95% CI [−5.89, −3.60], *P* < .00001) (Fig. 5A). In addition, 14 studies evaluated the neural functional deficit score according to NIHSS.^13^,^14^,^18^,^19^,^22^,^23^,^24^,^25^,^26^,^27^,^28^,^29^,^33^,^36^ The result displayed that compared with conventional treatment, XNJi could remarkably improve the neurological function in patients with acute cerebral hemorrhage (MD = −4.45, 95% CI [−5.49, −3.41], *P* < .00001) (Fig. 5B).

### 3.6 Serum level of hs-crp

**Figure 6:**
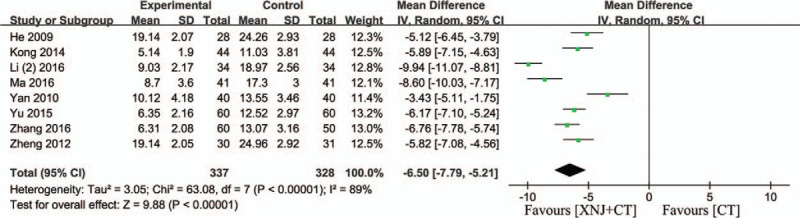
Forest plot of the hs-crp index. *I* ^*2*^ and *P* are the criterion for the heterogeneity test, ♦ pooled mean difference, —▪— mean difference and 95% CI. GCS = Glasgow Coma Scale.

Eight studies assessed serum hs-crp in patients.^15^,^23^,^24^,^27^,^36^,^37^,^38^,^39^ As shown in Fig. 6, there was a substantial heterogeneity (*P* < .00001, *I* ^2^ = 89%), and therefore the random-effect model was used. The result demonstrated that XNJi could significantly reduce the serum level of hs-crp compared with conventional therapy in patients with acute cerebral hemorrhage (MD = −6.50, 95% CI [−7.79, −5.21], *P* < .00001) (Fig. 6).

### 3.7 GCS index

**Figure 7:**
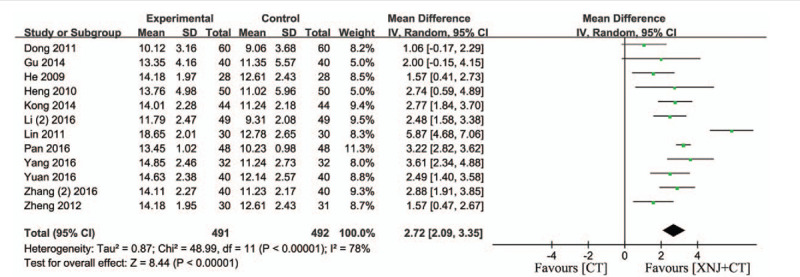
Forest plot of the serum level of GCS. *I* ^*2*^ and *P* are the criterion for the heterogeneity test, ♦ pooled mean difference, —▪— mean difference and 95% CI.

Twelve studies employed the GCS to evaluate prognosis for patients.^17^,^18^,^19^,^23^,^25^,^26^,^27^,^28^,^29^,^33^,^38^,^39^ The *I* ^2^ statistic showed that there was a significant heterogeneity among 12 trials (*I* ^2^ = 78%, *P* < .00001), and random-effect model was used to pool the result of these trials. Data analysis showed that compared with the conventional treatment, XNJi was able to remarkably improve the score of GCS in patients with acute cerebral hemorrhage (MD = 2.72, 95% CI [2.09, 3.35], *P* < .00001) (Fig. 7).

### 3.8 Activities of daily living

**Figure 8:**
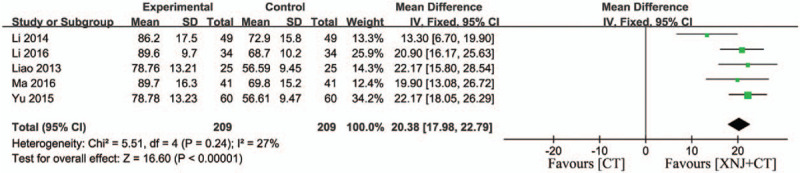
Forest plot of the activities of daily living. *I* ^2^ and *P* are the criterion for the heterogeneity test, ♦ pooled mean difference, —▪— mean difference and 95% CI.

Five studies adopted ADL score to evaluate prognosis for patients. There was no significant heterogeneity (*P* = .24, *I* ^*2*^ = 27%) and a fixed-effect model was used.^12^,^22^,^24^,^31^,^36^ The result indicated that XNJi significantly increased the scores of ADL compared with conventional treatment in patients (MD = 20.38, 95% CI [17.98, 22.79], *P* < .00001) (Fig. 8).

### 3.9 Cerebral hematoma volume

**Figure 9:**
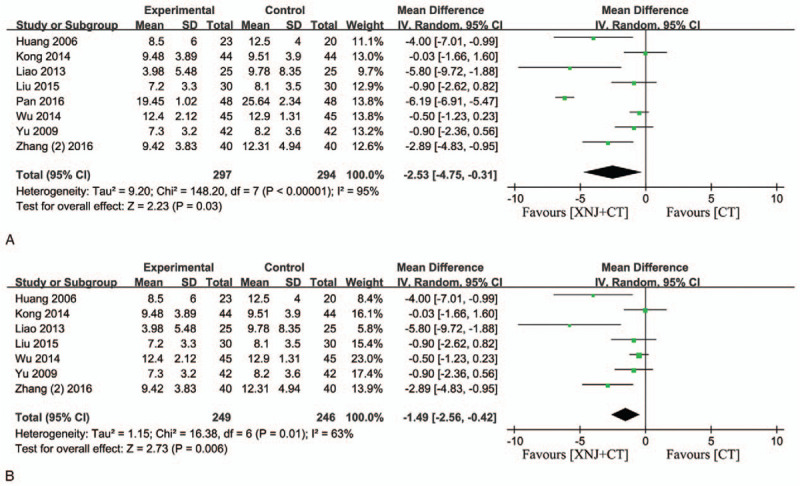
Forest plot of cerebral hematoma volume. (A) Forest plot of cerebral hematoma volume of all the 8 studies; (B) the forest plot of cerebral hematoma volume except the study “Pan 2016”. *I* ^2^ and *P* are the criterion for the heterogeneity test, ♦ pooled mean difference, —▪— mean difference and 95% CI.

Eight of the enrolled studies evaluated the hematoma volume of patients.^11^,^14^,^21^,^23^,^25^,^28^,^31^,^35^ The *I* ^*2*^ showed that there was significant heterogeneity among these 8 trials (*I* ^*2*^ = 95%, *P* < .00001) and random-effect model was used to pool the result. The result revealed that XNJi was able to significantly reduce the cerebral hematoma volume compared with the conventional treatment (MD = −2.53, 95% CI [−4.75, −0.31] *P* = 0.03(Fig. 9A). In addition, the sensitivity analysis showed that the study “Pan 2016” might be the main impact of heterogeneity. After carefully comparing with other included studies, it indicated that there was difference of treatment course between pan 2016 and other studies (10 days and 14–21 days, respectively). Therefore, the length of treatment course might be the main generation of heterogeneity (Fig. 9B).

### 3.10 Cerebral edema

**Figure 10:**
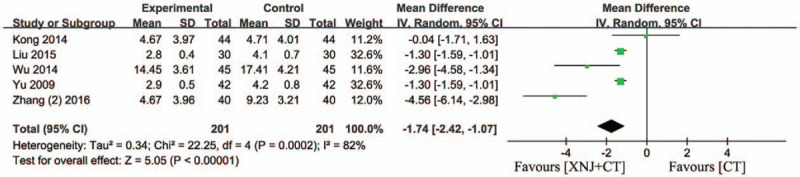
Forest plot of cerebral edema. *I* ^*2*^ and *P* are the criterion for the heterogeneity test, ♦ pooled mean difference, —▪— mean difference and 95% CI.

Five studies assessed the cerebral edema. There was significant heterogeneity among 5 trials (*I* ^*2*^ = 82%, *P* = .0002) and random-effect model was applied.^11^,^14^,^23^,^25^,^35^ The result demonstrated that XNJi significantly reduced the cerebral edema in patients with acute cerebral hemorrhage compared with conventional treatment (MD = −1.74 95% CI [−2.42, −1.07] *P* < .00001) (Fig. 10).

### 3.11 Bias analysis

**Figure 11:**
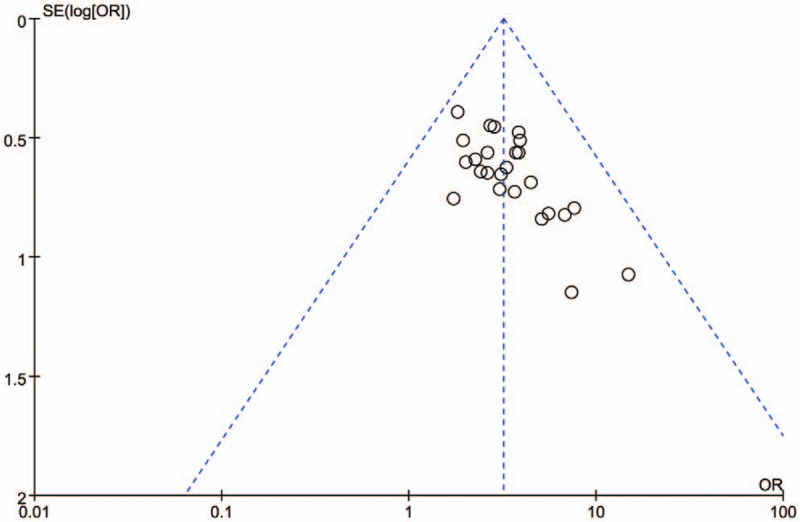
Funnel plot of XNJi with conventional treatment versus conventional treatment on efficacy. XNJi = Xingnaojing injection.

Funnel plot was used to assess the publication bias of included studies (Fig. 11). In this analysis, the funnel plot was asymmetric, suggesting that potential publication bias might affect the result. This publication bias might be related to the small sample size and quality of included studies.

## 4 Discussion

Acute cerebral hemorrhage is a common cerebral vascular disease with high mortality and disability rate. Till now, there is no specific treatment at home and abroad. The main clinical manifestations were headache, dizziness, confusion, coma, movement, and language barrier.^[40]^ Recent studies have suggested that hematoma enlargement was one of the most important causes of neurological deterioration. Cerebral edema can be caused by coagulation and surrounding brain tissue within minutes after intra-cerebral hemorrhage. The formation of cerebral edema is one of the most important causes of the structural and functional damage in nerve system after acute cerebral hemorrhage.^[41]^ Moreover, hematoma continues to expand as the first cause of neurological deterioration after 3 hours.^42^,^43^ After the hematoma formation and expansion, mechanical compression injury and ischemic changes occurs and results in a series of pathological changes.

In recent years, XNJi is widely used for acute cerebral hemorrhage. Researches have shown that the bioactive compounds of XNJi are germacrone, curdione, β-elemene, Camphor, curcumenol, muscone, (+) – borneol, (-) – borneol, and so on.^[44]^ It is commonly applied with a range of dose and period for the effect of reducing the blood brain barrier permeability, alleviating hydrocephalus, scavenging free radicals, promoting patient recovery, shortening coma time, as well as reducing complications in cerebral diseases. Meanwhile, XNJi is reported as an efficient agent for accelerating construction of collateral circulation, increasing capillary network and reducing vascular pressure in hemorrhage site. Several studies report that XNJi and conventional therapy combined with therapies such as decreasing blood pressure, maintaining water and electrolyte balance, and neuroprotective agent. It also developed the systematic analysis of the studies to confirm the value of treating acute cerebral hemorrhage.^45^,^46^

The results from our meta-analysis indicated that applying XNJi combined with conventional therapy could enhance the total response rate in patients with acute cerebral hemorrhage. In terms of short-term improvement on neurological impairment, daily activities of patients, coma status, inflammatory level, hematoma volume and cerebral edema volume, treatment group was also superior to control group. It could be speculated from the systemic analysis that XNJi might get superior efficacy to conventional therapy in reducing patient inflammatory level, hematoma volume, and cerebral edema volume, as well as in promoting patient consciousness and mobility recovery.

The result of the analysis indicated that there was potential efficacy of XNJi on patients with acute cerebral hemorrhage. It demonstrated improvement of CSS, NHISS and impairment of hs-crp, hematoma, and edema compared with conventional treatment. Moreover, in our research, the appropriate time for XNJi on acute cerebral hemorrhage in the course ranged from 1 hour to 5 days. XNJi got remarkable efficacy at the dose of 20, 30, 60 mL and from 7 to 28 days. In addition, no serious adverse reactions occurred. Therefore, it might provide a potential therapeutic option for patients. Especially, several large-scale, high quality and reasonable design plan, strict follow-up, and randomized uniform criteria are needed to verify its ideal application of dosage and course.

## 5 Conclusion

The systematic review and meta-analysis indicated that XNJi was effective in treating acute cerebral hemorrhage with significant decrease of neurologic impairment and no serious adverse reactions. The results suggested that XNJi can be used accompany with conventional treatment at the dose of 20, 30, 60 mL and from 7 to 28 days from the course of 1 hour to 5 days. The meta-analysis of this research was based on several small-sample and few high-quality studies. Therefore, studies with rigorous, large-scale RCTs of XNJi in treating acute cerebral hemorrhage were further needed to confirm its efficacy, safety, and detailed characteristic of application.

## Acknowledgments

The authors wish to thank reviewers for their critical comments provided during revision and also wish to thank all authors of references.

## Author contributions

XM, TW and JXW performed the search and wrote the manuscript. TW and YXY analyzed the data. YXY, TW and JW performed the data extraction. XM, WJZ, NZ and JW designed the study and amended the paper.
